# Measuring the context of care in an Australian acute care hospital: a nurse survey

**DOI:** 10.1186/1748-5908-5-60

**Published:** 2010-08-02

**Authors:** Timothy J Schultz, Alison L Kitson

**Affiliations:** 1 Australian Patient Safety Foundation, Playford Building, University of South Australia, Adelaide, South Australia, Australia; 2 Discipline of Nursing, School of Population Health and Clinical Practice, University of Adelaide, Adelaide, South Australia, Australia; 3 Centre for Evidence-Based Practice South Australia, a Collaborating Centre of the Joanna Briggs Institute, University of Adelaide, Adelaide, South Australia, Australia; 4 Green Templeton College, University of Oxford, Woodstock Road, Oxford, UK

## Abstract

**Background:**

This study set out to achieve three objectives: to test the application of a context assessment tool in an acute hospital in South Australia; to use the tool to compare context in wards that had undergone an evidence implementation process with control wards; and finally to test for relationships between demographic variables (in particular experience) of nurses being studied (n = 422) with the dimensions of context.

**Methods:**

The Alberta Context Tool (ACT) was administered to all nursing staff on six control and six intervention wards. A total of 217 (62%) were returned (67% from the intervention wards and 56% from control wards). Data were analysed using Stata (v9). The effect of the intervention was analysed using nested (hierarchical) analysis of variance; relationships between nurses' experience and context was examined using canonical correlation analysis.

**Results:**

Results confirmed the adaptation and fit of the ACT to one acute care setting in South Australia. There was no difference in context scores between control and intervention wards. However, the tool identified significant variation between wards in many of the dimensions of context. Though significant, the relationship between nurses' experience and context was weak, suggesting that at the level of the individual nurse, few factors are related to context.

**Conclusions:**

Variables operating at the level of the individual showed little relationship with context. However, the study indicated that some dimensions of context (*e.g*., leadership, culture) vary at the ward level, whereas others (*e.g*., structural and electronic resources) do not. The ACT also raised a number of interesting speculative hypotheses around the relationship between a measure of context and the capability and capacity of staff to influence it.

We propose that context be considered to be dependent on ward- and hospital-level factors. Additionally, questions need to be considered about the unit of measurement of context in studies of knowledge implementation--is individual (micro), ward (meso) or hospital-level (macro) data most appropriate? The preliminary results also raise questions about how best to utilise this instrument in knowledge translation research.

## Background

In 1998, Kitson *et al*. [1] defined context as the environment or setting in which people receive healthcare services or, in relation to evidence implementation, 'the environment in which the proposed change is to be implemented.' The importance of context in shaping the effectiveness of knowledge implementation has been acknowledged [2-4]. Put simply, context matters [5] and implementation strategies that work in one setting may not work in a different setting with different context.

The role of context has been defined in the PARIHS framework (Promoting Action on Research in Health Services), which hypothesised that successful implementation (SI) of evidence into clinical practice occurs as a function (f) of the scientific robustness of the evidence (E), the receptiveness of the context (C) of the care setting and the appropriateness of the facilitation (F) of the change process [1]. Consequently, SI = f (E, C, F) [6]. Context can be conceptualised as a continuum ranging from 'weak' to 'strong' [7].

Initially, context was considered to be dependent on three sub-elements: leadership, culture, and measurement [1,8]. The features of positive leadership include clear role delineation, the promotion of teamwork, staff autonomy, and effective organisational structures in which everybody is a leader of something [1,7]. Culture has been commonly defined as 'the way things are done around here' [9], while Bate's description states that an organisation's culture is not something that it 'has,' rather it is 'something an organisation is' [10]. McCormack *et al*. [7] summarise the features of a strong culture as: the ability to define culture in terms of prevailing beliefs; valuing of individual staff and clients; promotion of learning; and consistency of values around relationships, teamwork, power and authority, and rewards/recognition. The final sub-element 'measurement' has since been re-defined as 'evaluation,' to capture multiple methods of monitoring and feedback processes, including peer review, user-led feedback, and reflection on practice [7,8]. A strong evaluation context involves feedback at individual, team, and system levels, and captures information from a range of sources [7].

Although the study of context and research uptake is relatively new, relationships between stronger context, greater research utilisation, and less staff and patient adverse events in nursing have been documented [5,11]. Most initial work in this field has examined how factors that operate at the individual (micro), unit (meso), and organisational (macro) level influence research uptake. For example, Estabrooks *et al*. [12] found that individual level variables (such as time spent on the internet, and lower levels of emotional exhaustion) were positively related to research utilisation and explained more variation than either unit level (*e.g*., context, nurse-to-nurse collaboration) or organisational level (*e.g*., hospital size) factors.

Despite the theoretical advancements of the PARIHS framework, few studies have quantified the sub-elements to context (culture, leadership, and evaluation) or have considered how external factors--operating at micro, meso or macro levels--determine context.

### Measuring context

Two tools to measure context--both based on the PARIHS framework--have recently been developed: the Context Assessment Index (CAI) and the Alberta Context Tool (ACT). The CAI, developed by Brendan McCormack and researchers at the Universities of Ulster and Cork in Ireland, is both an evaluative and self-assessment tool [13]. Users are encouraged to monitor the state of the context into which they wish to introduce a new innovation or piece of evidence [13]. The ACT was developed by the Knowledge Utilisation Studies Program (KUSP) at University of Alberta to measure organisational context for five different types of healthcare workers (nurses, doctors, allied health, clinical specialists, and managers) [14]. The ACT adds eight additional dimensions to the three previously defined elements of context (leadership, culture, and evaluation, which is termed 'feedback processes' in the ACT). Broadly, these additional dimensions relate to organisational slack (having a buffer or cushion of actual or potential resources with respect to time, space, and human resources, *e.g*., 'how often do you have 'down time"), structural and electronic processes (elements that facilitate the ability to access and use research, *e.g*., 'how often do you use the library'), and information-sharing activities (informal and formal organisational structures that make research use more probable, *e.g*., 'how often do you interact with people in the following roles') [14]. The tool was piloted in four hospitals and subsequently refined and subjected to psychometric testing. A total of 30 items from the questionnaire were removed from the draft questionnaire based on low response rate, poor correlation with dimensions of context and principal components analysis; factor analysis indicated a sound structure accounting for 70% of the variance of organisational context; internal reliability for the dimensions was verified with Cronbach's alpha scores ranging from 0.65 to 0.92, considered to be acceptable for new scales [14].

### The TOPIC7 project

The Older Person and Improving Care (TOPIC7) project was set up in a tertiary acute hospital in South Australia. The Nursing Department in the hospital had, since 2005, undertaken a cycle of audits of its standards of nursing care. These audits showed that certain elements of care were not improving. The TOPIC7 project was therefore proposed to improve the experience of older people going through the acute hospital sector by using a Knowledge Translation (KT) toolkit. This clinical initiative provided an opportunity to explore the links between patient safety, quality improvement (QI) and evidence-based practice (EBP) approaches along with an exploration of how knowledge translation could be linked with these techniques. In addition, the initiative was seen to operationalise in a very practical way the policy priority of the state around improving older peoples' care [15].

Results of the actual intervention study have been reported elsewhere [16,17]. The focus of this paper is on the way context was measured as part of the TOPIC7 study, and to examine whether context varied at sites exposed to the TOPIC7 intervention compared to control sites.

### Aims and objectives

The ACT had not previously been used outside of Canada. Therefore, the first aim of this project was to test whether the ACT was culturally applicable and suitable for use in an Australian acute care hospital. The second aim was to compare context between hospital wards involved in the evidence implementation (*i.e*., intervention wards) and control sites. The third aim was to investigate what factors impact on context and to determine the presence or absence of variables that could be targeted to strengthen context and improve the uptake of evidence into practice, as indicated by the PARIHS framework.

## Methods

### Design and sample

The study employed a cross-sectional design to examine context in a large tertiary acute care hospital in South Australia. Six hospital wards that were included in the TOPIC7 implementation were compared with six control wards picked at random from the remaining hospital wards. Context was measured between October and November 2008, corresponding to the tail end of the 12-month TOPIC7 implementation phase. Logistical constraints prevented measurements before the implementation. The context evaluation was conducted alongside of, but independent to, the TOPIC 7 implementation. Further detail of the site and evidence implementation is provided elsewhere [16,17]. The project was approved by the hospital's Human Ethics Research Committee (Protocol No:080609).

### Data collection

KUSP provided access to the ACT (nursing). Following a review by a group of five multidisciplinary health researchers, the terminology used in the ACT was slightly modified. For example, we used the term 'enrolled nurse' in place of 'licensed practical nurse,' and added a range of other positions including 'clinical nurse' and 'clinical service coordinator.'

The ACT has 73 items, 14 relating to demographics and site issues and 59 relating to the 11 dimensions of context, which are summarised in Table 1. Dimensions 1 to 3, 6, 8 to 10 are calculated as the mean response to typically six questions using a 5-point Likert scale ranging from 'strongly disagree' to 'strongly agree.' for the 'leadership' dimension, questions included 'looks for feedback even when it is difficult to hear' and 'effectively resolves conflicts that arise.' Dimensions 4, 5, 7, and 11 summarise responses to questions about the frequency of events. For these dimensions, responses to each question (for example 'how often do you have time to talk to someone about new clinical knowledge') is dichotomised to 0 (for 'never,' 'rarely' or 'occasionally') or 1 (for 'frequently' or 'almost always') and a total sum is calculated for each dimension [14]. In all cases, a higher score is indicative of a more positive, or stronger, context.

**Table 1:** Summary of 11 dimensions of the context of acute care nursing.

Dimensions of context	Range	Control		Intervention			
		**mean**	**SD**	**mean**	**SD**	**Treatment effect (P)**	**Ward effect (P)**
**1. Leadership**	1-5	3.8	0.8	3.7	0.8	0.75	0.002
**2. Culture**	1-5	3.9	0.6	3.8	0.6	0.48	0.001
**3. Feedback processes**	1-5	3.4	0.8	3.1	0.9	0.14	0.000
4. Information sharing interaction	0-7	2.7	1.9	2.6	1.8	0.74	0.22
5. Information sharing activities	0-5	2.3	1.6	2.1	1.4	0.62	0.05
**6. Information sharing social processes**	1-5	4.0	0.6	4.0	0.5	0.59	0.06
7. Structural and electronic resources	0-11	3.9	2.8	3.2	2.6	0.07	0.78
**8. Organisation slack - Human resources**	1-5	3.1	1.1	2.8	1.0	0.36	0.000
**9. Organisation slack - Space**	1-5	2.7	1.0	2.6	1.0	0.43	0.000
**10. Organisation slack - Time**	1-5	3.0	0.6	2.9	0.6	0.44	0.000
11. Organisational slack	3-15	8.8	2.1	8.3	2.0	0.25	0.000

Means and standard deviations are presented for control and intervention wards. For all dimensions, the number of valid responses varied from 94 to 96 (control) and 119 to 128 (intervention). Dimensions in **bold **are calculated as mean values, those in normal font as the sum of dichotomised (0 or 1) items.

Ward leaders were briefed about the selected tool, and the least disruptive time period for the evaluation was selected. All nursing staff (enrolled nurses and registered nurses) listed on the payroll for each of the 12 wards were sent a copy of the questionnaire and an explanatory document via the hospital's internal mail. After five weeks, non-responders were sent another copy of the questionnaire. Questionnaires could be returned to the research team either by a collection box stationed in the ward, or by the hospital internal mail.

### Data analysis

Questionnaires were entered into an Excel spreadsheet and imported into Stata (v9, StataCorp, Texas, USA) software. Depending on data type and underlying distribution, a range of statistical tests were used to compare the demographics of the two groups, including chi-square, t-test, and Mann-Whitney U-tests. To account for the likely dependence of data collected within wards, hierarchical (nested) ANOVA was used to test for differences in the dimensions of context between control and intervention sites [18].

We conducted canonical correlation analysis (CCA) to test for relationships between the dimensions of context and staff experience. Staff experience was measured using two self-reported variables: time (months) in their current position (*i.e*., as a registered nurse, or enrolled nurse), and time (months) that they have worked on their current ward/unit. Given the relative infrequency of CCA in nursing literature, it is informative to briefly introduce the method and the calculated statistics. CCA involves the derivation of a number of independent canonical functions that maximise the correlation between linear composites (canonical variates), which are sets of dependent and independent variables [19]. The maximum number of canonical functions is equal to the number of variables in the smaller data set--in this case there were two variables in the 'experience' canonical variates, therefore two functions were derived. The CCA calculates the maximum amount of shared variation between the two canonical variates in the first function, then the second function is calculated by maximising the remaining unexplained variance [20]. The strength of the relationship between the canonical variates is calculated by the canonical correlation coefficient (R_c_), analogous to the multiple R in regression. Similarly the squared canonical correlation coefficient () is analogous to R^2 ^in regression and indicates the proportion of variance explained by the two canonical variates [21]. The significance of all canonical functions considered together, and any individual functions, may be tested using a range of tests including Wilks' lambda [20,21].

Standardised canonical function coefficients and structure coefficients (r_s_) of each canonical function are routinely reported in CCA [21]. Standardised canonical function coefficients are analogous to weights in regression analysis and are indicative of the contribution a variable makes to predicting, or explaining, the composite of variables in its set [19]. The r_s _represents the correlation between an observed variable and the calculated canonical function that describes the relationship between the two canonical variates [19]. Sherry and Henson [21] consider an r_s _of greater than 0.45 or less than -0.45 to be of sufficient size to indicate an important relationship. The square of r_s _() indicates the proportion of variance an observed variable shares with the calculated canonical function and is analogous to other r^2 ^effect size statistics. The communality coefficient (h^2^) is the proportion of the variance explained by each variable across all canonical functions, and is calculated as the sum of  across each function [19].

Thematic analysis [22,23] was used to analyse text responses to an open-ended question included in the ACT that asked nurses to outline the activities they would like to undertake if they had more time to spend on their ward.

## Results

### Questionnaire response rate

In the first round, 422 questionnaires were sent out and 165 completed questionnaires were returned. Additionally, 69 questionnaires were 'returned to sender,' indicating that staff were on extended leave or had left the ward. The second round yielded a further 52 completed questionnaires for a final response rate of 61.5% (217/353) appropriately addressed questionnaires. The response rate was significantly greater at intervention sites (66.9%, 121/181) than at control sites (56%, 96/172) (χ^2 ^= 4.5, df = 1, P = 0.033). The response rate at the ward level varied from 6% (at a control ward where the collection box was inexplicably lost) to 86% at an intervention ward (median 69%).

### Demographics

The demographics of participants at the control and intervention sites were similar (Table 2). Overall, 80% of participants were female. There was no difference in the age structure of participants from control or intervention sites (χ^2 ^= 9.2, df = 8, P = 0.42) and overall, less than half (41%) of participants were aged 40 or more (Table 2). Similarly, there were no differences in the make-up of staff positions (χ^2 ^= 9.1, df = 6, P = 0.17). Registered nurses made up the bulk (56.7%) of the participants, followed by enrolled nurses (21.0%) and associate clinical service coordinators (7.1%). In terms of highest qualification, there was no difference between the control or intervention sites (χ^2 ^= 2.9, df = 3, P = 0.41), with most participants (59.7%) having a Bachelor degree. The proportion of staff who had undertaken a specialist course was at borderline significance (50.5% in the control versus 36.1% in the intervention group, P = 0.051); although there was no difference in the proportion currently enrolled in an educational program or between experience as measured by time worked in current position or at the current site (Table 2).

**Table 2:** Summary of participants' demographics from control and intervention sites.

Demographic	Variable	Control	Intervention	Statistic	P
Gender	% Female	81.3	79.7	3.2^A^	0.21
Age	% 40 or over	37.6	42.9	0.39^A^	0.53
Staff position	% RN	52.1	60.2	9.1	0.17
	% EN	21.9	20.3		
	% Assoc Clinical Service Coordinators	8.3	6.3		
Highest qualification	% Diploma/Certificate	36.3	40.0	2.9^B^	0.41
	% Bachelor	60.4	59.2		
	% Masters	2.2	0.0		
	% PhD, DN	1.1	0.8		
Specialist courses	% completed	50.5	36.1	3.8^A^	0.051
Currently enrolled	% enrolled	22.6	19.8	0.10^A^	0.75
Length in current position	Median months	60	57	-1.08^C^	0.28
Length working on ward	Median months	56	36	1.05^C^	0.29
Hours worked last week	Mean hours	34.9	34.3	0.45^D^	0.65
Employment status	% Full time	47.9	49.6	0.02^A^	0.089

Statistics presented include ^A ^chi-square, ^B ^Fisher's exact test, ^C ^Mann-Whitney U-test and ^D^ Student’s t-test

### Context of care

The effect of the intervention on each of these dependent variables was examined using nested analysis of variance, with sites nested in the treatment group (control or intervention). Demographic variables (gender, primary role, length of time in current position, length of time working on unit, and hours worked in the last week) were tested for potential confounding. Based on comparison of means between treatment groups, plotting residuals and comparison of different ANOVA models, two independent variables in particular (namely length of time in current position and length of time on unit) were considered to be potential confounders for many of the dimensions of context, in particular leadership and culture. However, comparison of models with and without the potential confounders revealed very similar results, hence the simpler models excluding the confounders are presented here.

**Figure 1:**
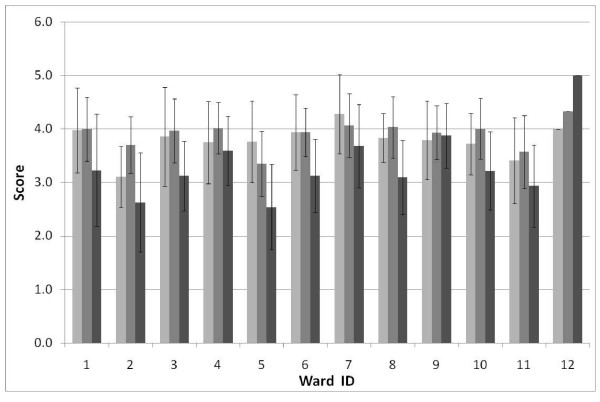
**Mean ± 1 SD for three dimensions of context**. Leadership (light), Culture (medium) Feedback processes (dark) across 12 wards (wards 1 to 6 intervention sites; wards 7 to 12 control).

**Figure 2:**
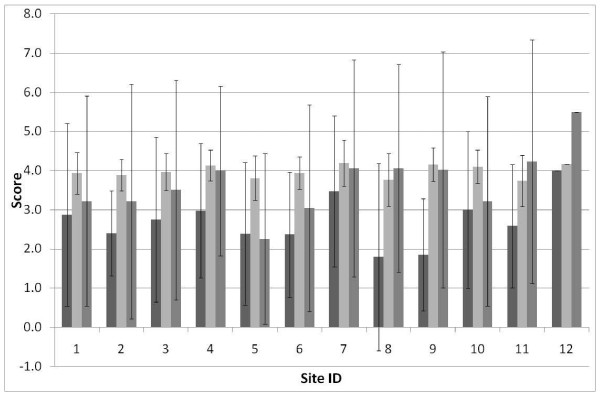
**Mean ± 1 SD for three dimensions of context**. Information sharing interaction (light), information sharing social processes (medium), structural and electronic resources (dark) across 12 wards (wards 1 to 6 intervention sites; wards 7 to 12 control).

The results indicated that there was no difference in any of the dependent variables between the treatment groups (Table 1); similarly, none of the demographic variables accounted for significant variation in the ANOVA (P > 0.05) and these variables were therefore removed from the model. Importantly, the ANOVA showed that for a large number (eight) of the 11 dimensions the nesting factor (ward) was highly significant, indicating significant variation between wards (Table 1). As shown in Figure 1, the three core dimensions derived from the PARIHS framework--leadership, culture, and feedback processes--all varied significantly between wards. 'Information sharing interactions,' 'Information sharing social processes' and 'structural and electronic processes' were the only dimensions that did not vary significantly between wards (Figure 2). The SD around these mean values for these three dimensions tended to be greater than for the other eight dimensions that did show significant variation between sites (Table 1, Figure 1, Figure 2).

Comparison of the dimensions of context is complicated because of their different derivations and scoring ranges. Examination of only those dimensions calculated as means (shaded in Table 1) indicates that across all wards, 'Information sharing social processes' scored highest, followed by 'culture,' 'leadership,' and 'feedback processes.' The three 'organisational slack' dimensions, in particular 'time,' tended to score the lowest.

These findings are supported by the fact that 83% of respondents (180/217) felt that more time would be useful. A total of 276 comments to the open-ended question were collated (Table 3). In particular, respondents identified the need for more time to undertake fundamental aspects of patient care (88/276 responses), especially routine care (54 responses). Prioritising more time to undertake information retrieval and research related activities was identified by 40/276, or 21% of responses.

**Table 3:** Summary of responses to open-ended question 'What I would do with more time?'

Theme	Specific areas	total
Fundamentals of Care	Have more time to discuss patient's condition (with them) (11)	88
	Do extra things for patients (8)	
	Do routine things properly *e.g*., washing showering (54)	
	Contact time with family (10)	
	Providing psychological, emotional, spiritual care (5)	
Learning and Teaching	Professional development (19)	57
	Clinical presentations by experts (6)	
	Educating junior staff (28)	
	Taking better care of myself (2)	
	Advanced clinical skills (2)	
Specific Clinical Aspects	Assessment (6)	42
	Patient education (19)	
	Counselling (6)	
	Discharge planning (5)	
	Patient involvement (5)	
	Palliative care (1)	
Information retrieval	More time to do more in-depth searching for information (40)	40
Staffing Issues/infrastructure	Night duty staff challenges (1)	25
	Keeping equipment clean/tidy (6)	
	Nursing documentation (9)	
	Non-nursing duties (1)	
	Better handovers/orientation (4)	
	More down time/recovery time (4)	
Research	Ward based research (13)	17
	QI/protocol work (4)	
Teamwork	More time to communicate with colleagues (6)	6
Total		276

The second largest group of responses coalesced around learning and teaching activities. The emerging picture from these open-ended responses is one of a nursing workforce challenged to find the time to undertake the core aspects of its role. Such a perception, real or otherwise, is bound to have an effect on the attitudes, behaviours and beliefs of the cohort of workers, particularly if they are being requested to spend more of an already scarce resource on innovations.

### Canonical correlation analysis

The canonical correlations (R_c_) were 0.39 and 0.34, equivalent to  of 0.15 and 0.12, respectively. Overall, both canonical functions were significant (Wilks λ = 0.75, F_20,370 _= 2.87, P < 0.0001), however, the variance shared between the variate sets, which is calculated as (1 - Wilks λ) and is equivalent to the overall effect size, is relatively low at 25%. The second canonical function on its own was significant (F_9,186 _= 2.66, P = 0.0063); however, shared variance was low (11%).

The standardised canonical function coefficients and structure coefficients for canonical functions one and two are presented in Table 4. Examination of these statistics must be considered in light of the relatively low effect sizes mentioned above. The data indicate that variables 'structural and electronic resources' and 'culture' exhibited the largest impact on context in function one, as these coefficient scores (0.724 and 0.715) were greatest. These results were mostly reflected when examining the structure coefficients, which indicated that 'organisational slack - human resources' and 'organisational slack - space' also impacted on the canonical function. Of the two experience variables, 'months on current unit' exhibited slightly greater influence over the variable composite and the canonical function. Because the experience variables 'culture' and 'structural and electronic processes' are all positive integers, they are positively related. Staff that had more experience tended to rate these aspects of context ('culture' and 'structural and electronic resources') more highly.

**Table 4:** Canonical correlation analysis for context assessment and nurse's experience for F1 and F2.

	1			2			
Variable	Coef	**r**_**s**_	**(%)**	Coef	**r**_**s**_	**(%)**	**h**^**2**^**(%)**
Leadership	0.072	0.252	6.4	0.160	0.329	10.8	17.2
Culture	0.715	0.473	22.4	0.211	0.405	16.4	38.8
Evaluation (feedback processes)	-0.128	0.213	4.5	-0.427	0.053	0.3	4.8
Information sharing interactions	-0.165	-0.054	0.3	-0.119	0.443	19.6	19.9
Information sharing activities	-0.555	-0.181	3.3	0.779	0.864	74.6	77.9
Information sharing social processes	-0.372	-0.097	0.9	-0.149	0.341	11.6	12.6
Structural and electronic resources	0.724	0.297	8.8	0.347	0.679	46.1	54.9
Organisational slack - Human resources	0.442	0.420	17.6	0.013	0.127	1.6	19.3
Organisational slack - Space	0.200	0.382	14.6	-0.045	0.093	0.9	15.5
Organisational slack - Time	-0.524	-0.093	0.9	0.223	0.369	13.6	14.5
			15			12	
Months in current position	0.453	-0.824	67.9	-1.11	-0.566	32.0	99.9
Months on current unit	0.677	-0.926	85.7	0.986	0.379	14.4	100.1

'Coef' = standardised canonical function coefficient; r_s _= structure coefficient;  = squared structure coefficient; h^2 ^= communality coefficient;  = squared canonical correlation coefficient. Structure coefficients and communality coefficients greater than 0.45 and 45%, respectively, are underlined.

With respect to the second function, 'information sharing activities' was the main context variable contributing; additionally, 'structural and electronic resources' also added explanatory power to the function. Both of the experience variables, in particular the 'months in current position,' exerted a strong impact on the experience composite. The relationship between 'months in current position' and 'information sharing activities' was negative, suggesting that as the length of time spent by nurses in a position increased, their participation in 'information and sharing processes' decreased.

Examination of the communality coefficients across both functions indicates: the very high overall relevance of both of the experience variables; the most important of the context variables were 'Information sharing activities' and 'Structural and electronic processes'; and most of the remaining context variables (in particular 'evaluation,' 'information sharing social processes,' 'organisational slack - space,' and 'organisational slack - time') did not contribute to the variation between experience and context, and thus can be interpreted as being invariant of experience of nursing staff.

The redundancy index is a measure that allows calculation of the shared variance explained by each canonical function [20]. For both dependent and independent variates, it is calculated as the product of the mean of  and the squared canonical correlation coefficient (, also included in Table 4). For function one, the redundancy index is (0.154 × 0.08 = 0.012), and for function two it is (0.114 × 0.196 = 0.022).

## Discussion

### Using the ACT to measure context in an Australian acute care setting

The ACT was applicable in a cultural setting (Australia) that is somewhat different to that in which it was developed, suggesting that the tool may have wide acceptance across the English-speaking developed world, at least. The tool is currently being used across four European countries [24] in a process that will inform the tool's utility across other languages. Moreover, we found a high response rate (61.5%) in our setting, using a hardcopy questionnaire with follow up of non-responders. This response rate compares favourably with the results of ACT pilot testing that found a response rate of 43% using both online and hardcopy versions of the tool with reminder letters across four hospitals [14]. Therefore, we consider that the tool was well received by Australian nurses. This may partly be related to a greater propensity to respond to the survey at intervention wards, probably because nurses there were more familiar with the TOPIC 7 project. Low response rates occurred in two control wards--due to the unexplained loss of a collection box (6% response rate), and the perception of 'push back' from another ward's leader (17% response rate) who did not actively support the research process. These findings indicate that good response rates are achievable with this tool but site preparation is important.

In contrast, the tool that was not selected for use (the CAI tool) is specifically devised to act as both a self assessment and an evaluative tool. While it is still in the early stages of development and refinement, there are a number of important issues to be discussed around how such a tool can be used as a reliable repeat measure, and whether it can complement the ACT approach. Indeed, the question would still need to be asked as to whether these two tools are measuring the same concepts at all.

Despite these questions, both tools operationalise the complex concepts around context, and through further refinement and use may be able to locate key indicators or predictors of successful innovation. There is clear potential for using these tools for diagnostic purposes to identify wards/sites in which context is amenable to change. Alternatively, dimensions of context may be targeted. For example, overall in this hospital it would appear that 'feedback processes' lag behind 'leadership' and 'culture.' Knowledge translation facilitators could address this issue as the first stage of evidence implementation.

### Variation in context between and within wards

This study detected significant variation between wards in many of the dimensions of context, suggesting several important issues for future work on this topic. While factors such as patient acuity and specialism, patterns of care, work method, and physical layout of the ward probably exert an influence, we feel that the role of ward leaders in developing culture, maintaining appropriate feedback mechanisms, and influencing organisational slack is more likely to be important [25]. This suggests that targeted interventions to strengthen these aspects of context should be based at the level of wards and ward leaders. Research needs to incorporate factors that operate at the ward level (such as leadership experience and style). Further, measurement and analysis of dimensions of context need to take into account ward-level variation and the requirement for hierarchical analysis of any data [18].

Other dimensions of context, chiefly the 'information sharing' dimensions and 'structural and electronic resources' did not vary between wards (P ≥ 0.05). While it is possible that these dimensions are inherently more variable, or the items used to measure the dimensions are less precise, it seems likely that these dimensions of context operate at the level of the hospital. Therefore, measurements across multiple wards are not required to accurately assess these dimensions of context in a hospital. Although Estabrooks' study [14] found no difference in these dimensions of context between hospitals, their results were combined across five different healthcare professions, and comparisons for each profession between hospitals were not made.

Finally, the lack of a difference between intervention and control wards is perhaps not surprising, given the exploratory nature of the study and the different approaches of the interdisciplinary teams involved [16]. The assessment of context in this study has generated baseline measurements that will be of value in future studies of context in acute care and in particular has raised the question of whether the ward needs to be the unit of analysis rather than individuals when considering context.

### Context in acute care nursing

Aggregation of data from the present study and the original study of Alberta nurses allows a simple comparison of context between Australian and Canadian hospitals (Table 5). The ratios of mean Australian:Canadian scores ranged from 1.03 to 1.14, indicating slightly higher scores in the Australian hospital, Mean scores were slightly greater in the Australian nurse data, as indicated by ratios. However, the median score of one dimension--structural and electronic resources--was greater in Alberta, a pattern that was reversed for organisational slack. These findings suggest greater accessibility of information for Canadian nurses, but far less time and space to use such resources compared to Australian nurses. However, these findings are only preliminary. The Canadian study reported differences between the four hospitals for many context dimensions (combining all professions together), indicating the inherent variability in context between hospitals in the same country and suggesting that meaningful comparison between countries would require a more integrated study.

**Table 5:** Summary of data from all wards from this study (South Australia, n ≅ 217) compared to data obtained from Alberta nurses in the KUSP study (n ≅ 152), including the ratio of the two mean scores.

Dimensions of context	Measure	South Australia		Alberta		Ratio
			SD		SD	
**1. Leadership**	Mean	3.8	0.8	3.6	1.0	1.06
**2. Culture**	Mean	3.9	0.6	3.8	0.7	1.03
**3. Feedback processes**	Mean	3.2	0.9	2.8	0.9	1.14
4. Information sharing interaction	Median	3		2		
5. Information sharing activities	Median	2		2		
**6. Information sharing social processes**	Mean	4.0	0.5	3.83	0.6	1.05
7. Structural and electronic resources	Median	3		5		
**8. Organisation slack - Human resources**	Mean	2.9	1.1	NR		
**9. Organisation slack - Space**	Mean	2.6	1.0	NR		
**10. Organisation slack - Time**	Mean	2.9	0.6	NR		
11. Organisational slack	Median	8.7		5.5		

Dimensions in bold are calculated as mean values, those in normal font as the sum of dichotomised (0 or 1) items.

NR = not reported

### Measuring context

This study raises a series of questions around what is the appropriate unit of measurement for context and how context may be potentially altered. The ACT demonstrated significant variation of key dimensions between wards (*i.e*., at the unit, or meso-level), whereas other dimensions were consistent across wards and therefore did not vary at the hospital, or macro-level. Our study did not indicate any readily measurable variables at the level of the individual nurse (*i.e*., the micro-level). Therefore, we propose that context be considered to be dependent on ward- and hospital-level factors, and that efforts to improve poor or weak context need to address both meso- and macro-levels.

### CCA analysis

The relationship between nursing staff members' experience and the context of care in their work environment is not particularly strong--as evidenced by the small redundancy indices and small effect sizes from the CCA. This supports our earlier finding that context of nursing in acute care hospitals is more strongly related to meso- and macro-level factors. Differences in context between wards are apparently due to factors that operate at the level of the ward, not individual nursing staff within the ward.

While CCA is infrequently used in nursing research, its availability in modern statistical software packages such as SPSS and Stata has placed it well within the reach of researchers in all disciplines. As a multivariate analytical technique, it can reduce the likelihood of Type I errors by eliminating the need for numerous multiple regression analyses when multiple data sets are examined [21]. Additionally, incorporating multiple causes and effects can better reflect the realities of human behaviour and complex systems than using singular variables [21]. Our use of CCA has provided insight into both its strengths and weaknesses. A strength is that it allows combination of related variables--for example, the 11 dimensions of context--that would otherwise be difficult to consider as there is no current method of calculating a total 'context' score [14]. However, a weakness, as shown here, is that although the overall model was highly significant, the effect sizes for both functions were relatively small, contributing to weak relationships between canonical variates in this study.

### Limitations

The limitations to this study include the use of a tool in a different culture to which it was developed. While every effort was made to make relevant healthcare and cultural modifications to the tool; these modifications were not rigorously tested.

Equally, it could be argued that the trialling of the two tools (ACT and CAI) should have been undertaken by clinicians rather than researchers with a specialist knowledge of evidence-based practice and knowledge translation. Their assessment of the useability of either tool may not have been the same as clinicians

Additionally, as previously mentioned, it would be imprudent to ascribe any cause and effect between the intervention and outcome measurements because no baseline measurements were made, and the study was only conducted at a single point in time. Other limitations include the unexplained loss of completed questionnaires from a control site, the very low response rate from one ward and the greater response rate at intervention wards compared to control wards. All of the above limitations can be addressed in subsequent applications of the tool.

## Conclusions

The study provoked as many questions as it answered, both from a conceptual design perspective and from a methodological perspective. Acknowledging the limitations of the study we conclude with the following recommendations from the study:

1. The ACT is acceptable for use in Australian hospitals for nurses with only minor modifications.

2. We need a better understanding of dimensions of context that do not apparently differ between wards and that may be more variable at a higher level (*i.e*., the hospital).

3. The fact that many of the context dimensions varied between wards affects how measurements of context can be accurately made and interpreted. Experimental design should allow measurements across a number of units/wards and analysis using hierarchical models.

4. The factors that shape context at the ward level are presumably related to interdependence between context dimensions such as leadership, culture, feedback processes and organisational slack. Interventions seeking to strengthen context in hospitals should consider the benefits of focussing at this level (*e.g*., improving ward leaders' leadership skills) rather than continuing to explore individual nurse characteristics in isolation (for example level of experience and training).

## Competing interests

The authors declare that they have no competing interests.

## Authors' contributions

TS conducted the evaluation, its statistics, and drafted the first version of the manuscript. AK designed and oversaw the implementation project, led the comparison of the CAI and ACT tools and analysed the open-ended question. Both authors designed the evaluation, and the study results and key findings were jointly interpreted by both authors. Both authors contributed to subsequent and final drafts of the manuscript, and take responsibility for the study findings.
